# Safety Assessment of *Sophora flavescens* Root Extract for Cosmetic Use: An Integrated Approach Using In Vitro, In Silico MoS, TTC, and History of Safe Use

**DOI:** 10.3390/toxics14050398

**Published:** 2026-05-06

**Authors:** Sangwon Gil, Hogeon Lee, Seung Ha Lee, Seung A. Shin, Dal Woong Choi, Kyung-Min Lim

**Affiliations:** 1 College of Pharmacy and Graduate School of Pharmaceutical Sciences, Ewha Womans University, Seoul 03760, Republic of Korea; swkil514@naver.com; 2 Transdisciplinary Major in Learning Health Systems, Department of Health and Safety Convergence Science, Korea University, Seoul 02481, Republic of Korea; geon2014_n@naver.com (H.L.); km1004sh@korea.ac.kr (S.H.L.);; 3 Graduate Program in Innovative Biomaterials Convergence, Ewha Womans University, Seoul 03760, Republic of Korea

**Keywords:** *Sophora flavescens* root extract (SFRE), threshold of toxicological concern (TTC), history of safe use (HSU), margin of safety (MoS), genotoxicity

## Abstract

Evaluating the safety of botanical extracts for cosmetics has become mandatory, but it is often challenging because of their phytochemical complexity and limited toxicological data. In this study, the safety of aqueous *Sophora flavescens* root extract (SFRE), widely used in cosmetics, was assessed using an integrated approach combining in vitro, in silico, margin of safety (MoS), threshold of toxicological concern (TTC), and history of safe use (HSU). Chemical characterization was performed by literature review and LC–MS/MS analysis. SFRE was classified as non-irritant in in vitro skin and eye irritation tests conducted according to OECD TG439 and 492. Whole-extract and constituent-level in silico analysis and literature evaluation were conducted to assess genotoxicity and skin sensitization potential. For systemic toxicity, a 13-week oral repeat dose no-observed-adverse-effect level (NOAEL) of 10 mg/kg bw/day for a decocted *Sophorae radix* extract was employed without compositional adjustment to calculate the acceptable systemic exposure dose of 0.10 mg/kg bw/day, which was slightly lower than the current usage of SFRE in cosmetics (up to 0.13 mg/kg/day). The TTC approach revealed that many bioactive constituents fell outside the applicability domain due to steroid moieties. HSU data from dietary supplements (32–64.67 mg/kg/day) could support the safety of the current use of SFRE in cosmetics. The findings highlight that a combined, case-by-case application of MoS, TTC, and HSU is essential for the robust safety assessment of complex botanical ingredients.

## 1. Introduction

*Sophorae flavescentis* radix is the dried root of *Sophora flavescens* Aiton (Fabaceae) and has long been used in East Asian traditional medicine. It has been described for the treatment of gastrointestinal, dermatological, and inflammatory conditions, and its major bioactive constituents include quinolizidine alkaloids and prenylated flavonoids. In particular, matrine- and oxymatrine-type alkaloids are regarded as characteristic constituents of this medicinal plant [1].

The pharmacological and medicinal relevance of *Sophora flavescens* has been recognized in official monographs in several countries. In China, it is listed in the Pharmacopoeia of the People’s Republic of China, and in Korea, it is included in the Korean Herbal Pharmacopoeia [2].

In recent years, *S. flavescens*-derived materials have also attracted attention as cosmetic ingredients. According to the European Commission CosIng database, *Sophora flavescens* root extract is defined as the extract of the roots of *Sophora flavescens* and is used as a skin-conditioning ingredient. In addition, use entries for *Sophora flavescens*-derived ingredients are available in cosmetic ingredient inventories such as ChinaCosIng [3].

The diverse biological activities of *S. flavescens* are attributed to the abundance of secondary metabolites present in its root. Liu et al. [4] reported that *S. flavescens* contains abundant quinolizidine alkaloids, including matrine, oxymatrine, and sophocarpine, and these constituents are considered major contributors to its biological activity. In addition, flavonoid compounds such as kurarinone, kuraridin, and kushenol D have been identified and are known to exert potent antioxidant and anti-allergic effects.

Although *S. flavescens* has a long history of use as a food and medicinal material with well-documented efficacy, a safety assessment that can clear potential toxicological concerns associated with dermal exposure from the use of cosmetics remains unconducted. This study aimed to conduct a safety assessment for aqueous *Sophora flavescens* root extract (SFRE) as a cosmetic ingredient, applying three approaches. Margin of safety (MoS)-based estimation was used to calculate acceptable exposure levels using existing toxicological literature data from comparable botanical materials. The threshold of toxicological concern (TTC) approach was used to derive systemic exposure thresholds based on the identified chemical constituents and their respective Cramer classifications [5,6], and the history of safe use (HSU) approach was used to calculate the safe daily exposure level from dietary use. In addition, evaluations of local and pivotal toxicological endpoints, including skin irritation, eye irritation, skin sensitization and genotoxicity, were conducted in vitro or in silico to ensure overall safety. By integrating these methodologies, this study presents a case study to apply non-animal test-based safety assessment methodologies applicable to botanical ingredients.

## 2. Materials and Methods

### 2.1. Preparation of Sophora flavescens Root Extract

The *Sophora flavescens* root extract (SFRE) used in this study was manufactured and supplied by The Garden of Natural Solution Co., Ltd. (Osan-si, Republic of Korea). The extract was produced according to a conventional cosmetic raw material manufacturing process, and a literature review was conducted to determine appropriate pretreatment conditions.

Briefly, 10 kg of dried *S. flavescens* root was extracted with 100 L of distilled water at 80 °C. To enhance extraction efficiency, ultrasonic extraction was performed for 30 min, followed by an additional 30 min of shaking extraction. The extract was then concentrated using a rotary vacuum evaporator, followed by freeze-drying and pulverization to obtain a powdered extract. The final extraction yield (plant-to-extract ratio) was approximately 22.15%.

The quality specifications of the extract were verified based on appearance (brown powder), odor (characteristic odor), heavy metal content [lead (≤10 ppm) and arsenic (not detected)], and microbiological limits (not detected).

### 2.2. Experimental Characterization of SFRE Constituents (Chromatography-Based Analysis)

The chemical composition of SFRE was characterized through literature review and experimental analysis. For the literature survey, publicly accessible databases, including the United States Department of Agriculture (USDA) database, PubMed, and SciFinder, were utilized. Experimental analyses of SFRE were conducted using standard food composition analysis methods and LC–MS/MS.

Analyses of nutritional components, including moisture, fat, protein, carbohydrates, and trace components, were performed by the Korea Food Research Institute (KFRI) in accordance with official analytical methods specified in the Korean Food Standards Code [7].

#### LC-MS/MS Analytical Conditions

The reference compounds, Matrine, Oxymatrine, and Sophoridine, were purchased from TCI Korea (Seoul, Republic of Korea). Kurarinone, Formononetin, and Sophocarpine were purchased from Merck (Darmstadt, Germany). Daidzein, Genistein, and Cytisine were purchased from Thermo Fisher Scientific Korea Ltd. (Seoul, Republic of Korea).

The liquid chromatographic–mass spectrometric analysis was performed using the following instrumental parameters (Table 1)and multiple reaction monitoring conditions to detect each reference compound are as shown in Table 2.

### 2.3. Cramer Classification and TTC Grouping

The chemical constituents identified through LC–MS/MS analysis and literature review were classified into Cramer Classes I–III using Toxtree^®^ v3.1.0 (Joint Research Centre, European Commission). The SMILES structural representations of each compound were obtained from PubChem and entered into the Decision Tree for Cramer Classification module.

As a result, genistein and daidzein were classified as Cramer Class III following a conservative approach.

#### In Silico Genotoxicity Prediction of Bacterial Reverse Mutagenicity for the Chemical Constituents Contained in SFRE

The constituents identified in SFRE and the chemical structures of each compound were entered into ChemTunes^®^ in SMILES format [8]. Predictions of potential mutagenicity were primarily based on existing data from bacterial reverse mutation tests (Ames tests). In cases where such data were unavailable, results from in vitro chromosome aberration tests or in vivo micronucleus assays were used to infer mutagenic potential. For constituents lacking all relevant experimental data, mutagenicity was predicted using in silico methods implemented in ChemTunes^®^.

### 2.4. Calculation of Systemic Exposure Dose (SED)

To estimate consumer exposure, a leave-on cosmetic product containing 2.126% SFRE, corresponding to the maximum use concentration listed in China COSING [3], was assumed. The systemic exposure doses (SEDs) for individual constituents were estimated according to the SCCS Notes of Guidance for the Testing of Cosmetic Ingredients and Their Safety Evaluation, 12th revision [9]. For aggregate exposure assessment, a conservative default cosmetic exposure of 269 mg/kg bw/day was applied [9]. In the absence of SFRE-specific dermal absorption data, a default dermal absorption value of 100% was applied in accordance with SCCS guidance [9]. This value was used as a conservative screening assumption for the estimation of systemic exposure.

For constituent-level exposure estimation, concentrations of individual constituents were derived from the experimentally measured composition of the present SFRE whenever quantitative analytical data were available. Specifically, oxymatrine, matrine, sophoridine, kurarinone, sophocarpine, and cytisine were quantified in the present extract and used directly for SED estimation. For identified constituents lacking quantitative analytical data, a conservative default concentration of 1 ppm in the finished product was assumed for screening-level exposure estimation to avoid underestimation of potential exposure.

The resulting SED values were then compared with relevant TTC thresholds, taking into account each constituent’s Cramer classification and genotoxicity alert status, where applicable.

### 2.5. In Silico Assessment of Skin Sensitization Using Derek Nexus

Each dataset was processed in Derek Nexus v.6.5.1 using Derek Knowledge Base (KB) 2025 1.0. If a skin sensitization alert was activated with a reasoning level of certain/probable/plausible/equivocal, the chemical was considered to be a sensitizer. Chemicals activating an alert with a reasoning level of doubted/improbable, or chemicals not activating any alerts, were considered to be non-sensitizers.

The interpretation of Derek Nexus reasoning levels followed the convention that alerts with certain, probable, plausible, or equivocal confidence were treated as positive alerts, whereas doubted or improbable alerts and no-alert outputs were treated as negative for screening purposes [10].

### 2.6. In Vitro Skin Irritation Test with a 3D Reconstructed Human Epidermis Model

In this study, the reconstructed human epidermis model KeraSkin™, developed using a “me-too” approach based on OECD Test Guideline 439, was employed [11]. KeraSkin™ was supplied by Biosolution Co., Ltd. (Seoul, Republic of Korea), and the DMEM-based culture medium was also provided by the same supplier. KeraSkin™ tissues were placed in 6-well plates containing 0.9 mL of culture medium per well and pre-incubated at 37 °C under 5% CO_2_ for 22 ± 2 h. After pre-incubation, 40 μL of the test substance was applied directly onto the tissue surface. The insert was gently tilted using sterile forceps to ensure even distribution of the test substance across the tissue surface.

Dulbecco’s phosphate-buffered saline (DPBS) was used as the negative control, while 5% sodium dodecyl sulfate (SDS) dissolved in DPBS was used as the positive control. The SFRE sample was prepared as a 50% (*w*/*v*) solution in DPBS. Following application, the plates were incubated at 37 °C under 5% CO. After 30 min of exposure, the tissues were rinsed with DPBS and subsequently subjected to a post-incubation period of 42 ± 2 h, after which the culture medium was completely removed.

### 2.7. In Vitro Eye Irritation Test with 3D Reconstructed Human Corneal Epithelium Model

An in vitro eye irritation test was conducted using the MCTT HCE™ model, a multilayered reconstructed human corneal epithelium composed of basal, wing, and squamous cells, developed in accordance with OECD Test Guideline 492 and supplied by Biosolution Co., Ltd. [12]. For three-dimensional reconstruction, the cells were cultured on 12 mm diameter Millicell^®^ inserts (Millipore, Burlington, MA, USA) under air–liquid interface conditions for 7 days to regenerate a 3D human corneal epithelium.

The models were shipped to the test facility under refrigerated conditions in a 24-well plate format, placed on agarose gel. The culture medium was pre-warmed at 37 °C for 30 min prior to use. For pre-incubation, 900 μL of the pre-warmed medium was added to each well of a 6-well plate, and the HCE model inserts were carefully transferred into the wells. The tissues were then pre-incubated at 37 °C under 5% CO_2_ for 22 ± 2 h. To perform the test, 40 μL of the test solution was topically applied to the apical epithelial surface (0.6 cm^2^) of each model insert following pre-incubation. The tissues were exposed to the test substance for 10 ± 1 min at 37 °C under 5% CO_2_. After exposure, the tissues were rinsed to remove residual test substance and further incubated for an additional 16 ± 1 h.

DPBS was used as the negative control, and methyl acetate was used as the positive control. The SFRE sample was prepared as a 20% (*w*/*v*) solution in DPBS. Tissue viability was assessed using the WST-1 assay [Water-Soluble Tetrazolium Salt-1; 2-(4-iodophenyl)-3-(4-nitrophenyl)-5-(2,4-disulfophenyl)-2H-tetrazolium sodium salt; CAS RN 150849-52-8].

### 2.8. Estimation for History of Safe Use

To provide contextual information on prior human exposure relevant to history of safe use (HSU), food consumption data and marketed dietary supplement information were collected from publicly available sources. Food consumption information relevant to exposure assessment was obtained from the EFSA Comprehensive European Food Consumption Database, which provides detailed consumption data across European populations and is widely used in dietary exposure assessment. In parallel, dietary supplements containing *Sophora flavescens* were identified using the Dietary Supplement Label Database (DSLD; https://dsld.od.nih.gov), developed by the Office of Dietary Supplements of the U.S. National Institutes of Health (NIH), which compiles label information for dietary supplement products marketed in the United States [13,14,15].

Representative products containing *Sophora flavescens* (Ku Shen; Non-Alcohol, label ID 238120) were identified in the DSLD, and the labeled serving size, recommended daily intake frequency, and dosage form were recorded. Where available, product label information on extract type, extract amount, or formulation characteristics was also noted. However, when the extraction solvent, extraction ratio, or constituent standardization was not clearly specified on the product label, no further quantitative back-calculation of extract-equivalent intake was performed. In such cases, the product information was used only as supportive contextual evidence of prior marketed human exposure and not as a basis for precise exposure estimation.

## 3. Results

### 3.1. Comprehensive Chemical Profiling of Sophora flavescens Root Extract

The chemical composition of *Sophora flavescens* Root Extract (SFRE) as a cosmetics ingredient was systematically investigated and identified. Through an extensive review of the Duke Phytochemical and Ethnobotanical Database of the United States Department of Agriculture (USDA) and relevant scientific literature, a total of 60 chemical constituents present in *S. flavescens* extract were identified.

**Figure 1:**
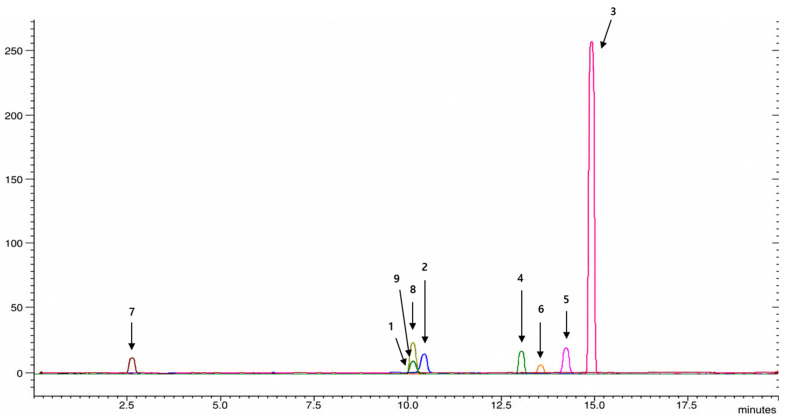
LC-MS/MS chromatogram of the authentic standards of major bioactives of SFRE: 1. Matrine, 2. Oxymatrine, 3. Kurarinone, 4. Daidzein, 5. Formononetin, 6. Genistein, 7. Cytisine, 8. Sophocarpine, 9. Sophoridine.

**Figure 2:**
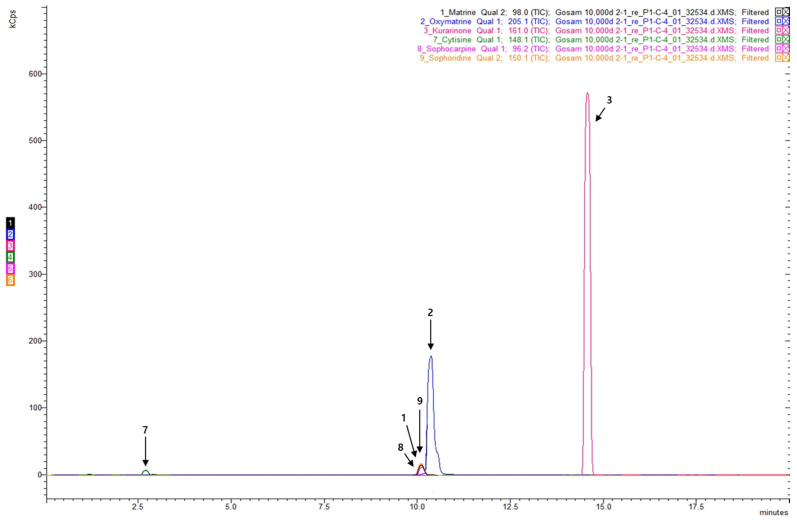
Chromatographic profiles of individual components in SFRE: 1. Matrine, 2. Oxymatrine, 3. Kurarinone, 7. Cytisine, 8. Sophocarpine, 9. Sophoridine.

Quantitative analysis of the chemical constituents of SFRE was performed from two perspectives: nutritional components and other bioactive constituents. Results from macro- and micronutrient and mineral analyses conducted by the Korea Food Research Institute indicated that 15 nutritional components accounted for more than 96% of the extract composition, with carbohydrates representing the largest fraction (68.9%). In addition, analysis of nine bioactive compounds for which reference standards were available identified oxymatrine, matrine, sophoridine, and kurarinone as major constituents of SFRE according to LC-MS/MS analysis (Figure 1 and Figure 2, see Section 2 for details).

More specifically, the nutritional analysis showed that SFRE was mainly composed of carbohydrates (68.9%), protein (17.7%), fat (2.2%), and water (4.7%), together with minor mineral constituents including potassium (1.372%), calcium (0.562%), magnesium (0.498%), phosphorus (0.421%), and sodium (0.074%). LC–MS/MS quantification further showed that oxymatrine (24.510 mg/g, 2.451%), matrine (3.331 mg/g, 0.333%), sophoridine (3.273 mg/g, 0.327%), kurarinone (2.139 mg/g, 0.214%), sophocarpine (1.163 mg/g, 0.116%), and cytisine (0.721 mg/g, 0.072%) were detected, whereas daidzein, formononetin, and genistein were not detected under the applied analytical conditions.

Overall, experimental analyses accounted for 99.97% of the total composition of SFRE, and detailed results are presented in Table 3. Experimental data were applied for realistic exposure scenarios, whereas maximum concentration values from the literature or experimental data were used for worst-case exposure scenarios to ensure a conservative safety evaluation.

### 3.2. Evaluation of Skin and Eye Irritation Potentials of Sophora flavescens Root Extract

**Figure 3:**
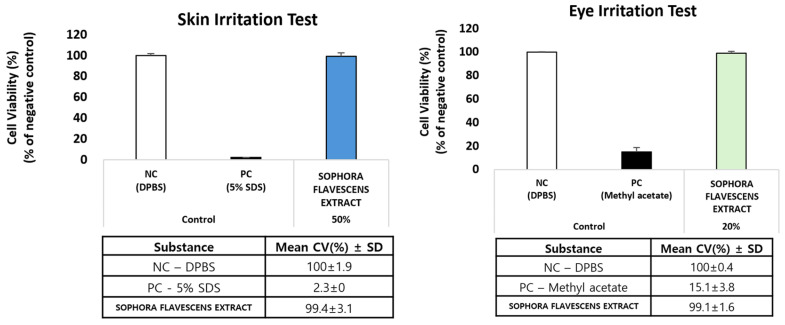
Evaluation of the skin and eye irritation potential of SFRE in reconstructed human epidermis (KeraSkin™) and reconstructed human corneal epithelium (MCTT HCE™) models. Classification was performed according to the decision criteria of OECD TG 439 and OECD TG 492. Data are presented as mean ± SD (n = 3).

The in vitro skin and eye irritation tests demonstrated that SFRE did not induce significant irritation in either the reconstructed skin epidermis or cornea models when tested at concentrations of 50% (*w*/*v*) and 20% (*w*/*v*), respectively. In both the reconstructed human epidermis model (KeraSkin™, a me-too test method of OECD TG439 [17]) and the reconstructed human corneal epithelium model (MCTT HCE™, a me-too test method of OECD TG492 [18]) (see Figure 3), SFRE was classified as non-irritant. In both assays, tissue viability values were well above the established threshold criteria for irritation classification. These findings indicate that topical application of SFRE is safe with respect to skin and eye irritation potential.

### 3.3. Genotoxicity Assessment

The genotoxic potential of *Sophora flavescens* root extract was previously evaluated by Che et al. [16] using a bacterial reverse mutation assay, an in vitro chromosome aberration assay, and an in vivo micronucleus assay. In that study, the bacterial reverse mutation assay was negative with and without metabolic activation, whereas the chromosome aberration assay suggested weak clastogenic activity under the test conditions. However, no significant micronucleus induction was observed in vivo. These findings provide informative extract-level evidence, but the available data remain limited and should be interpreted cautiously rather than as definitive evidence of the absence of genotoxic risk.

Importantly, the material used by Che et al. [19] was a decocted Sophorae radix extract, and compositional equivalence to the SFRE characterized in the present study has not been demonstrated. Therefore, the transferability of the published genotoxicity findings to the present extract remains uncertain and should be regarded as supportive rather than conclusive.

To strengthen the assessment, constituent-level information was additionally reviewed using in silico tools and the available literature. Fischer et al. [20] reported that neither matrine nor oxymatrine induced a significant increase in revertants in an OECD TG 471 bacterial reverse mutation assay [21], indicating that these two major quinolizidine alkaloids are unlikely to have gene mutation potential. However, direct experimental genotoxicity data for several other SFRE constituents remain limited, and positive or alerting in silico outputs for some flavonoid-related constituents could not be fully resolved.

Taken together, the currently available evidence does not allow a conclusion of a significant genotoxic concern under the proposed cosmetic use conditions. However, the database remains limited, and the absence of conclusive evidence should not be interpreted as proof of no risk.

### 3.4. In Silico Prediction of Skin Sensitization Potential of SFRE

The skin sensitization potential of 45 bioactive constituents identified in SFRE was evaluated using the Derek Nexus expert system used in OECD defined approach for skin sensitization [18]. Of the compounds assessed, 16 were predicted to be non-sensitizers. In contrast, 29 compounds triggered structural alerts, with 6 and 23 constituents classified as “equivocal” and “plausible” sensitizers, respectively. Potency estimations categorized the predicted sensitizers as follows: 10 weak, 3 moderate, 15 strong, and 1 extreme sensitizer.

A subset of 15 compounds was flagged as strong sensitizers based on the presence of substituted phenol analogs. However, a meticulous review of the underlying historical data revealed that this alert was triggered by inconsistent in vivo outcomes across various assays, including the local lymph node assay (LLNA) and the human repeat insult patch test (HRIPT). Notably, *o*-hydroxybenzoates (salicylates) and *p*-hydroxybenzoates (parabens)—despite their structural alerts—show a very low incidence of clinical sensitization in humans, even with widespread exposure in cosmetics [22,23]. The experimental data for these classes remain conflicting, often failing to demonstrate sensitization in standard animal models except under conditions of compromised skin barrier function [24].

Especially, sophocarpine was uniquely classified as an extreme sensitizer due to its α,β-unsaturated amide moiety. Theoretically, this structure allows for the formation of hapten–protein complexes via Michael addition at the β-carbon [25]. Nevertheless, the weight of evidence suggests that the electrophilic reactivity of such amides is significantly attenuated by resonance stabilization [26]. Representative analogs, such as acrylamide, are typically documented as weak or non-sensitizers in clinical and experimental settings [27]. Furthermore, many structurally related conjugated systems show no detectable sensitizing activity, indicating that the theoretical Michael addition potential does not necessarily translate to a high clinical risk [28].

Although in silico modeling identified several constituents of *S. flavescens* root extract as potential sensitizers, these compounds occur only at trace levels within the botanical matrix. Sophocarpine was detected at 0.116% of SFRE. When applying the most stringent dermal sensitization threshold (DST), 1.5 mg/cm^2^, for highly potent chemicals [29,30], (whole-body surface area is assumed as 17,500 cm^2^), this level fell well below this DST.

### 3.5. Estimation of Safe Usage Level of Aqueous Sophora flavescens Root Extract Based on Margin of Safety Approach

A margin of safety (MoS)-based assessment of systemic toxicity for SFRE was conducted based on repeated-dose oral toxicity data. Che et al. [19] and Kim [31] performed a 13-week repeated-dose oral toxicity study in rats with a decocted Sophorae radix extract and identified a no-observed-adverse-effect level (NOAEL) of 10 mg/kg bw/day.

For cosmetic safety assessment, a default uncertainty factor of 100 was applied to account for interspecies and interindividual variability. Accordingly, the acceptable systemic exposure dosage level derived from the published NOAEL was estimated to be 0.10 mg/kg bw/day.

Incidentally, the decocted Sophorae radix extract used in the subchronic toxicity study by Che [19] showed the total content of oxymatrine and matrine, representative quinolizidine alkaloids considered as the active component of *S. flavescens* extract [32], was 6.09%, which is about 2.19 fold higher than SFRE we are investigating (2.78%). This compositional difference between the decocted Sophorae radix extract and the SFRE used in the current study may be considered in correcting the NOAEL of SFRE; however, no toxicokinetic or dose–response data were available to justify a quantitative adjustment of the NOAEL. Therefore, the original published NOAEL was conservatively retained without correction.

### 3.6. Evaluation of TTC Applicability to the Chemical Constituents of SFRE

The constituents identified in SFRE were classified into Cramer Classes using Toxtree^®^ v3.1.0. In accordance with SCCS guidance and previous studies, TTC thresholds of 46 μg/kg body weight/day for Cramer Class I and 2.3 μg/kg body weight/day for Cramer Classes II and III were applied [8,33]. The classification results are summarized in Table 4.

Subsequent evaluation of TTC applicability indicated that TTC was not fully suitable for SFRE. Current TTC guidance states that substances with known endocrine-active effects should not be assessed using TTC, and that substances with suspected endocrine activity should also be considered on a case-by-case basis for applicability [8,34]. In addition, the Chinese TTC framework recommends that botanical mixtures should first be evaluated for TTC applicability, and that alternative safety assessment approaches should be used when constituents outside the applicability domain are present [35].

Based on these criteria, 24 flavonoid-related constituents in SFRE were conservatively considered to be outside the applicability domain of TTC. These 24 constituents consisted of three phytoestrogenic isoflavonoids (genistein, daidzein, and formononetin) and 21 prenylated or structurally related flavonoids. This distribution indicates that most of the constituents excluded from TTC applicability were flavonoid-related compounds with structural features potentially associated with endocrine-related activity. In particular, genistein, daidzein, and formononetin are well recognized as representative phytoestrogenic isoflavonoids [36], and this property may serve as a basis for limiting TTC applicability [37]. These isoflavonoids also exhibit significant hormonal and anticancer activities, representing a level of biological activity that is not well captured by the generic TTC framework originally developed for low-risk synthetic chemicals.

Meanwhile, among the remaining 21 non-food constituents, 17 were alkaloids, including matrine, oxymatrine, sophocarpine, sophoridine, cytisine, and structurally related quinolizidine alkaloids. Because these alkaloids constitute the major bioactive components of SFRE, they could formally remain within the TTC screening framework. However, this does not mean that they are low-concern substances [33]. Rather, Cramer Class III generally refers to compounds possessing structural features associated with relatively greater toxicological concern, and therefore does not fit well with the original purpose of TTC as a screening tool for low-risk chemicals.

Taken together, these findings indicate that TTC has only limited applicability for SFRE and is not suitable as the primary basis for deriving an acceptable use level. Therefore, in the present study, systemic safety was interpreted by integrating repeated-dose toxicity data, conservative point-of-departure (PoD) analysis, and history of safe human consumption.

### 3.7. History of Safe Use Approach

*Sophora flavescens* root extract has a long history of dietary consumption, and such extensive dietary exposure provides important evidence supporting its safety. In the present study, the safety of SFRE was further evaluated by investigating its regulatory status and dietary exposure levels in domestic and international contexts. In Korea, *S. flavescens* root is currently not listed as an edible ingredient in the “Food Standards and Specifications” administered by the Ministry of Food and Drug Safety [38,39]. However, international cases indicate that, in the United States, various dietary supplements containing *S. flavescens* root extract are commercially marketed and widely consumed.

NIH DSLD Ku Shen Non-Alcohol (ID: 238120): water-based maceration extraction (yield 0.34, 330 mg dried root → 970 mg liquid extract). NIH DSLD capsule products: hydroalcoholic extraction (1:4 ratio, 1.92 g dried root → 480 mg extract).

According to the NIH Dietary Supplement Label Database, products containing SFRE are commercially available in both liquid and capsule formulations. As shown in Table 5, liquid extract products are formulated to provide approximately 1320 mg/day of dried plant material, which corresponds to an intake of approximately 3880 mg/day of liquid extract. In addition, capsule-type dietary supplements typically contain 480 mg of SFRE per capsule and are recommended to be taken as two capsules twice daily, resulting in a total daily intake of approximately 1920 mg.

When dietary intake was converted to body weight-normalized exposure, the estimated acceptable exposure was approximately 64.67 mg/kg body weight/day for liquid formulations and 32 mg/kg body weight/day for capsule formulations, which was significantly higher than 0.10 mg/kg/day estimated with the MoS approach. Nevertheless, as oral ingestion and dermal application represent different exposure routes, potential differences in systemic absorption and toxicokinetic behavior should be considered as part of the overall safety assessment.

### 3.8. Exposure Level of Sophora flavescens Root Extract from Cosmetics

To estimate the systemic exposure dose (SED) of cosmetic ingredients, consumer exposure estimates by product category, as provided in the SCCS Guidance for the Testing of Cosmetic Ingredients and their Safety Evaluation [8], were applied. According to the guideline, the daily exposure from skincare products is approximately 180.04 mg/kg body weight/day, while leave-on skincare and haircare products correspond to approximately 225.82 mg/kg body weight/day. For the aggregated exposure scenario encompassing all cosmetic product types, a value of 269 mg/kg body weight/day has been proposed. In the present study, the most conservative exposure value of 269 mg/kg body weight/day was applied to estimate systemic exposure doses (SEDs).

According to the Inventory of Existing Cosmetic Ingredients in China [3] listed in ChinaCosIng, the maximum historical use level of *Sophora flavescens* root extract in leave-on cosmetic products was reported to be 2.126%, while use levels up to 8.3333% have been reported for rinse-off products. In this study, given the higher potential for systemic exposure associated with leave-on products, the more conservative use level of 2.126% for SFRE was selected as representative of the worst-case exposure scenario [3].

SED = Daily use amount (mg/kg) × C_product_/100 × Extraction yield/100 ÷ Plant: Extracting Solvent ratio × Skin absorption (%)/100

C_product_ = 2.126%, Extraction yield = 22.15%, Plant: Extracting Solvent ratio = 10, Skin absorption 100%

According to this equation, the systemic exposure of SFRE from cosmetics is about 0.13 mg/kg/day, which is slightly above the acceptable dose suggested by MoS (0.10 mg/kg/day) but within that derived from the history of safe use approach. This SED can be recalculated into exposure dose on the body surface area of 0.0074 mg/cm^2^/day (whole-body surface area is assumed as 17,500 cm^2^), which is far less than 1.5 mg/cm^2^, the most conservative dermal sensitization threshold for highly potent chemicals [30,40].

## 4. Discussion

Safety evaluation of botanical ingredients for cosmetic use should address local toxicity, genotoxicity, and systemic toxicity in accordance with international regulatory standards [8,41], using a case-by-case and weight-of-evidence approach, particularly when the test material is a chemically complex botanical extract with limited constituent-specific toxicological data. International regulatory frameworks likewise emphasize that botanical ingredients require structured safety assessment under intended use conditions and that the applicability of generic screening approaches should be considered carefully for complex mixtures.

In the present study, SFRE was experimentally confirmed to be non-irritant in both in vitro skin and eye irritation assays using validated reconstructed human tissue models, indicating a low potential for acute local irritation under the proposed conditions of use. In addition, skin sensitization concern was explored as supportive information at the constituent level using computational toxicology-based screening, although such outputs were interpreted conservatively and not used as stand-alone evidence. Taken together, the available local toxicity data support a low likelihood of acute topical irritation, while acknowledging that local tolerance assessment for botanical extracts should remain context-dependent.

With respect to genotoxicity, the present evaluation should be integrated as a weight-of-evidence assessment with important limitations. The published extract-level study by Che et al. [19] did not show significant micronucleus induction in vivo, but weak clastogenicity was observed in the in vitro chromosome aberration assay. Moreover, the tested material in that study was a decocted Sophorae radix extract, and compositional equivalence to the SFRE characterized in the present study has not been demonstrated. Therefore, the transferability of the published genotoxicity findings to the present extract remains uncertain and should be regarded as supportive rather than conclusive. To supplement the extract-level evidence, constituent-level information was additionally reviewed using in silico tools and the available literature. Fischer et al. [20] reported negative results for matrine and oxymatrine in a bacterial reverse mutation assay, suggesting that these two major quinolizidine alkaloids are unlikely to have gene mutation potential.

However, direct experimental genotoxicity data remain limited for several other SFRE constituents, and positive or alerting in silico outputs for some flavonoid-related constituents could not be fully resolved. For some flavonoid-related constituents, including genistein, the literature does not provide consistent evidence of mutagenicity and instead describes antioxidant and protective biological activities against genetic damage [36,42]. These literature findings are supportive, but they do not replace direct constituent-specific genotoxicity testing. Accordingly, the currently available evidence does not allow a conclusion of a significant genotoxic concern under the proposed cosmetic use conditions, but the database remains limited, and the absence of conclusive evidence should not be interpreted as proof of no risk.

Systemic toxicity interpretation also warrants caution. The original NOAEL of 10 mg/kg bw/day reported by Che et al. [19] was retained, and the corresponding acceptable systemic exposure was estimated as 0.10 mg/kg bw/day after application of the default uncertainty factor of 100. However, the adjustment of the NOAEL based on differences in marker constituents may be considered with further evidence, justifying a direct linear relationship between marker constituent concentration and systemic toxicity. Without this information, such an adjustment should be avoided [8] and the original NOAEL shall be taken conservatively.

The TTC-based constituent-level assessment also showed important limitations in the case of SFRE. A substantial number of flavonoid-related constituents, including isoflavonoid- or phytoestrogen-related compounds, were considered outside the applicability domain of TTC because of potential endocrine-related biological activity or structural characteristics that limit the use of generic TTC assumptions [34,35]. In particular, genistein, daidzein, and formononetin are well-recognized phytoestrogen-related isoflavonoids with biologically meaningful interactions in hormone-related pathways [36]. In addition, many of the remaining non-food-related constituents were assigned to Cramer Class III. These findings do not by themselves demonstrate risk, but they do indicate that TTC has only limited utility for a complex botanical mixture such as SFRE and should be interpreted as a screening-level tool rather than a definitive basis for safety conclusion.

Under the highly conservative aggregate exposure scenario applied in the present study, the estimated exposure to SFRE from cosmetics was slightly above the acceptable systemic exposure derived from the original NOAEL according to the conventional MoS-based approaches [43,44]. Therefore, the MoS-based interpretation alone provides limited assurance of the current usage of SFRE under worst-case assumptions. In this context, the history of safe human use and the absence of local toxicity may provide stronger supportive information, but they do not eliminate the uncertainty associated with limited extract-specific systemic toxicity or route-to-route difference in toxicity. The dietary history of *Sophora flavescens* may still be considered relevant as contextual evidence of prior human exposure, but it should not be interpreted as a full substitute for extract-specific toxicological evaluation for cosmetic use.

Overall, the present study supports a case-by-case, multi-evidence safety assessment strategy for SFRE rather than reliance on any single approach. The available data indicate low concern for acute local irritation toxicity, skin sensitization and systemic safety issues regarding the use of SFRE in cosmetics based on a history of safe use approach, but the potential genotoxicity and systemic toxicity issues could not be fully excluded because of limitations of information regarding extract compatibility and constituent-level data availability and the restricted applicability of TTC to this type of botanical mixture.

## 5. Conclusions

Aqueous *Sophora flavescens* root extract was confirmed to be non-irritant in the in vitro skin and eye irritation models used in the present study, and its dermal exposure is below the dermal sensitization threshold level. The overall safety assessment of SFRE was conducted using a multi-evidence approach integrating local toxicity data, in silico skin sensitization and genotoxicity assessment, genotoxicity literature data, constituent-level TTC screening, repeated-dose toxicity literature data, and dietary consumption history. Based on the totality of available evidence, SFRE is not considered to pose a significant safety concern under the proposed conditions of use, although some uncertainty remains.

At the same time, the present assessment indicates that the TTC framework has only limited applicability to complex botanical extracts such as SFRE. Several constituents were considered to fall outside the TTC applicability domain because of possible endocrine activity, while many other constituents were assigned to Cramer Class III on the basis of structural complexity. Accordingly, TTC alone does not provide a sufficient basis for the safety evaluation of SFRE.

For complex botanical ingredients such as SFRE, well-characterized extract-level toxicological data, particularly repeated-dose toxicity data, may provide a more scientifically appropriate basis for safety assessment when available. In the absence of such information, the history of safe human use through dietary intake may serve as supportive evidence in the overall interpretation of systemic safety. Further studies are nevertheless warranted to better characterize extraction-dependent differences in constituent composition, including marker constituent recovery, relative enrichment of major bioactive constituents, and compositional differences across extraction methods. Such data would further strengthen the scientific basis for the safety assessment of SFRE and other complex botanical ingredients intended for cosmetic use.

## Figures and Tables

**Table 1:** Test group composition and administration.

Parameters for liquid chromatography	Analysis method	LC-MS/MS		
	Column	AQUASIL C18 (150 mm × 3.0 mm, 3 μm)		
	Injection volume	1 μL		
	Mobile phase	A: 0.1% Formic acid in water B: MeOH		
	Flow rate	0.3 mL/min		
	Gradient	Time	A (%)	B (%)
		0	95	5
		1	95	5
		3	90	10
		5	40	60
		7	40	60
		8	10	90
		17	10	90
		18	95	5
		20	95	5
Parameters for mass spectrometry	Ionization	ESI (+/−)		
	Capillary voltage	4.0 kV (+), 4.5 kV (−)		
	Cone temperature	350 °C		

**Table 2:** Multiple reaction monitoring conditions for 9 kinds of target active constituents in SFRE.

No.	Chemicals	Cas No.	Polarity	Precursor Ion	Product Ion 1	Product Ion 2
1	Matrine	519-02-8	positive	249	148.10	98.00
2	Oxymatrine	16837-52-8	positive	265	205.10	247.10
3	Kurarinone	34981-26-5	negative	437	161.00	275.10
4	Daidzein	486-66-8	negative	253	208.00	132.10
5	Formononetin	485-72-3	positive	269	253.00	197.00
6	Genistein	446-72-0	positive	271	153.00	91.20
7	Cytisine	485-35-8	positive	191	148.10	44.40
8	Sophocarpine	6483-15-4	positive	247	96.20	179.00
9	Sophoridine	6882-68-4	positive	249	84.20	150.10

**Table 3:** Constituents of *Sophora flavescens* extract and their contents.

No	Chemicals	Chemical Class	Contents			Reference
			USDA (%)	Literature Value (%)	Experimental Value (%)	
1	Water	Inorganic			4.7	
2	Carbohydrates	Macronutrient			68.9	
3	Fat	Macronutrient			2.2	
4	Protein	Macronutrient			17.7	
5	Vitamin C	Micronutrient			0	
6	Vitamin E	Micronutrient			0	
7	Ca	Inorganic			0.562	
8	Na	Inorganic			0.074	
9	K	Inorganic			1.372	
10	Mg	Inorganic			0.498	
11	P	Inorganic			0.421	
12	Zn	Inorganic			0.002	
13	Fe	Inorganic			0.025	
14	Cu	Inorganic			0.001	
15	Mn	Inorganic			0.002	
16	1-Maakiain	Flavonoids	-			USDA
17	2′-Methoxykurarinone	Flavonoids	-	-		USDA [16]
18	5,7,4′-Trihydroxy-8-lavanduly-2′-methoxyflavanone	Flavonoids		-		[16]
19	8-Lavandulykaempferol	Flavonoids		-		[16]
20	8-Prenylkaempferol	Flavonoids		-		[16]
21	Citrusinol	Flavonoids		-		[16]
22	Cyclokuraridin	Flavonoids		-		[16]
23	Daidzein	Flavonoids			ND	
24	Demethylkuraridin	Flavonoids		-		[16]
25	Demethylxanthohumol	Flavonoids		-		[16]
26	Flavenochromane	Flavonoids		-		[16]
27	Formononetin	Flavonoids	-		ND	USDA
28	Genistein	Flavonoids			ND	
29	Isoanhydroicaritin	Flavonoids		-		[16]
30	Isokuraridin	Flavonoids		-		[16]
31	Isoxanthohumol	Flavonoids		-		[16]
32	Kosamol	Flavonoids		-		[16]
33	Kuraridine	Flavonoids		-		[16]
34	Kuraridinol	Flavonoids		-		[16]
35	Kurarinol	Flavonoids		-		[16]
36	Kurarinone	Flavonoids	-		0.214	USDA
37	Kushenol	Flavonoids		-		[16]
38	Leachianone	Flavonoids	-	-		USDA [16]
39	Noranhydroicaritin	Flavonoids		-		[16]
40	Norkurarinol	Flavonoids		-		[16]
41	Sophoflavescenol	Flavonoids		-		[16]
42	Sophoraflavanone	Flavonoids	-	-		USDA [16]
43	Xanthohumol	Flavonoids		-		[16]
44	7,11-Dehydromatrin	Alkaloids		-		[4]
45	7a-Hydroxysophoramine	Alkaloids		-		[4]
46	9a-Hydroxymatrine	Alkaloids		-		[4]
47	9a-Hydroxysophoramine	Alkaloids		-		[4]
48	Anagyrine	Alkaloids		-		[4]
49	Baptifoline	Alkaloids		-		[4]
50	Cytisine	Alkaloids		-	0.072	[4]
51	Kuraramine	Alkaloids		-		[4]
52	Lamprolobine	Alkaloids		-		[4]
53	Leontalbinine N-oxide	Alkaloids		-		[4]
54	Lupanine	Alkaloids		-		[4]
55	Mamanine	Alkaloids		-		[4]
56	Matrine	Alkaloids		-	0.333	[4]
57	Oxymatrine	Alkaloids		-	2.451	[4]
58	Sophocarpine	Alkaloids		-	0.116	[4]
59	Sophoranol	Alkaloids		-		[4]
60	Sophoridine	Alkaloids		-	0.327	[4]
	Total (%)		-	-	99.97	

ND, not detected.

**Table 4:** In silico safety assessment of identified constituents in *Sophora flavescens* root extract.

No	Cramer Class	Chemical	Classification	Genotoxicity	Skin Sensitization (Reasoning Level, EC3 Prediction)	TTC Threshold (μg/kg bw/d)	Leave-On Skin & Hair (SCCS)	269 mg/kg
							Realistic SED (μg/kg bw/d)	Worst-Case SED (μg/kg bw/d)
1	none	Water	Inorganic				268.7902	268.7902
2	none	Carbohydrates	Macronutrient				3940.3497	3940.3497
3	none	Fat	Macronutrient				125.8167	125.8167
4	none	Protein	Macronutrient				1012.2524	1012.2524
5	none	Vitamin C	Micronutrient				0.0000	0.0000
6	none	Vitamin E	Micronutrient				0.0000	0.0000
7	none	Ca	Inorganic				32.1404	32.1404
8	none	Na	Inorganic				4.2320	4.2320
9	none	K	Inorganic				78.4639	78.4639
10	none	Mg	Inorganic				28.4803	28.4803
11	none	P	Inorganic				24.0767	24.0767
12	none	Zn	Inorganic				0.1144	0.1144
13	none	Fe	Inorganic				1.4297	1.4297
14	none	Cu	Inorganic				0.0572	0.0572
15	none	Mn	Inorganic				0.1144	0.1144
16	III	Maackiain	Flavonoids	- (in silico)	Plausible (47%, weak)	2.3	0.0057	0.0057
17	OAD	2′-Methoxykurarinone	Flavonoids		Plausible (66%, weak)	2.3	0.0057	0.0057
18	OAD	5,7,4′-Trihydroxy-8-lavanduly-2′-methoxyflavanone	Flavonoids		Plausible (64%, weak)	2.3	0.0057	0.0057
19	OADIII	8-Lavandulykaempferol	Flavonoids		Plausible (0.34%, strong)	2.3	0.0057	0.0057
20	OAD	8-Prenylkaempferol	Flavonoids		Plausible (0.28%, strong)	2.3	0.0057	0.0057
21	III	Citrusinol	Flavonoids		Plausible (0.26%, strong)	2.3	0.0057	0.0057
22	OAD	Cyclokuraridin	Flavonoids		Plausible (10%, weak)	2.3	0.0057	0.0057
23	OAD	Daidzein	Flavonoids	- (Ames) + (in silico)	Equivocal (0.23%, strong)	2.3	0.0057	0.0057
24	OAD	Demethylkuraridin	Flavonoids		Plausible (8.7%, moderate)	2.3	0.0057	0.0057
25	OAD	Demethylxanthohumol	Flavonoids		Plausible (0.35%, strong)	2.3	0.0057	0.0057
26	III	Flavenochromane B	Flavonoids		Plausible (0.32%, strong)	2.3	0.0057	0.0057
27	OAD	Formononetin	Flavonoids	+ (in silico)	Plausible (0.25%, strong)	2.3	0.0057	0.0057
28	OAD	Genistein	Flavonoids	+ (Ames) + (in silico)	Plausible (0.25%, strong)	2.3	0.0057	0.0057
29	OAD	Isoanhydroicaritin	Flavonoids		Plausible (0.29%, strong)	2.3	0.0057	0.0057
30	OAD	Isokuraridin	Flavonoids		Plausible (9.1%, moderate)	2.3	0.0057	0.0057
31	OAD	Isoxanthohumol	Flavonoids		Equivocal (0.31%, strong)	2.3	0.0057	0.0057
32	III	Kosamol	Flavonoids		Plausible (71%, weak)	2.3	0.0057	0.0057
33	OAD	Kuraridine	Flavonoids		Plausible (9.1%, moderate)	2.3	0.0057	0.0057
34	OAD	Kuraridinol	Flavonoids		Plausible (10%, weak)	2.3	0.0057	0.0057
35	OAD	Kurarinol	Flavonoids		Plausible (75%, weak)	2.3	0.0057	0.0057
36	OAD	Kurarinone	Flavonoids		Plausible (63%, weak)	2.3	12.2385	12.2385
37	OAD	Kushenol	Flavonoids		Equivocal (0.36%, strong)	2.3	0.0057	0.0057
38	OAD	Leachianone	Flavonoids		Plausible (58%, weak)	2.3	0.0057	0.0057
39	OAD	Noranhydroicaritin	Flavonoids		Plausible (0.28%, strong)	2.3	0.0057	0.0057
40	OAD	Norkurarinol	Flavonoids		Plausible (72%, weak)	2.3	0.0057	0.0057
41	OAD	Sophoflavescenol	Flavonoids		Plausible (0.29%, strong)	2.3	0.0057	0.0057
42	OAD	Sophoraflavanone	Flavonoids		Equivocal (0.34%, strong)	2.3	0.0057	0.0057
43	OAD	Xanthohumol	Flavonoids	- (in silico)	Plausible (0.37%, strong)	2.3	0.0057	0.0057
44	III	7,11-Dehydromatrin	Alkaloids		Non-sensitizer	2.3	0.0057	0.0057
45	III	7a-Hydroxysophoramine	Alkaloids		Non-sensitizer	2.3	0.0057	0.0057
46	III	9a-Hydroxymatrine	Alkaloids		Non-sensitizer	2.3	0.0057	0.0057
47	III	9a-Hydroxysophoramine	Alkaloids		Non-sensitizer	2.3	0.0057	0.0057
48	III	Anagyrine	Alkaloids	- (in silico)	Non-sensitizer	2.3	0.0057	0.0057
49	III	Baptifoline	Alkaloids	- (in silico)	Non-sensitizer	2.3	0.0057	0.0057
50	III	Cytisine	Alkaloids	- (in silico)	Non-sensitizer	2.3	4.1176	4.1176
51	III	Kuraramine	Alkaloids		Non-sensitizer	2.3	0.0057	0.0057
52	III	Lamprolobine	Alkaloids		Non-sensitizer	2.3	0.0057	0.0057
53	III	Leontalbinine N-oxide	Alkaloids		Non-sensitizer	2.3	0.0057	0.0057
54	III	Lupanine	Alkaloids	- (in silico)	Non-sensitizer	2.3	0.0057	0.0057
55	III	Mamanine	Alkaloids		Non-sensitizer	2.3	0.0057	0.0057
56	III	Matrine	Alkaloids	- (in silico)	Non-sensitizer	2.3	19.0441	19.0441
57	III	Oxymatrine	Alkaloids	- (in silico)	Non-sensitizer	2.3	140.1712	140.1712
58	III	Sophocarpine	Alkaloids	- (in silico)	Equivocal (0.01%, extreme)	2.3	6.6340	6.6340
59	III	Sophoranol	Alkaloids	- (in silico)	Non-sensitizer	2.3	0.0057	0.0057
60	III	Sophoridine	Alkaloids	- (in silico)	Non-sensitizer	2.3	18.7009	18.7009

OAD, Out of Applicability Domain of TTC.

**Table 5:** Dietary intake level of *S. flavescens* root extract.

Country	Food Type	Processing	Serving Size or Composition	Consumption (Day)	Conversion to Extract Consumption (mg/kg/Day)
Republic of Korea	-	-	-	-	-
USA	Liquid	Extract (equivalent to 0.34 of dried plant material)	970 mg (equiv. 330 mg d.m.)	3880 mg (equiv. 1320 mg d.m.)	64.67
USA	Capsule	Extract	480 mg	1920 mg	32

## Data Availability

The original contributions presented in this study are included in the article. Further inquiries can be directed to the corresponding authors.

## Abbreviations

The following abbreviations are used in this manuscript:

TTC	Threshold of Toxicological Concern
SFRE	*Sophora flavescens* Root Extract
MoS	Margin of Safety
HSU	History of Safe Use
NOAEL	No-Observed-Adverse-Effect Level
PoD	Point of Departure
SED	Systemic Exposure Dose
SCCS	Scientific Committee on Consumer Safety
USDA	United States Department of Agriculture
OAD	Outside Applicability Domain
OECD	Organisation for Economic Co-operation and Development
DSLD	Dietary Supplement Label Database
NIH	National Institutes of Health
EFSA	European Food Safety Authority
KFRI	Korea Food Research Institute
DPBS	Dulbecco’s Phosphate-Buffered Saline
SDS	Sodium Dodecyl Sulfate
LLNA	Local Lymph Node Assay
HRIPT	Human Repeat Insult Patch Test
DST	Dermal Sensitization Threshold
